# A High-Quality Diet, as Measured by the DASH Score, Is Associated with a Lower Risk of Metabolic Syndrome and Visceral Obesity

**DOI:** 10.3390/biomedicines11020317

**Published:** 2023-01-23

**Authors:** Klaudia Konikowska, Wojciech Bombała, Andrzej Szuba, Dorota Różańska, Bożena Regulska-Ilow

**Affiliations:** 1 Department of Dietetics and Bromatology, Wroclaw Medical University, Borowska Street 211, 50-556 Wroclaw, Poland; 2 Statistical Analysis Center, Wroclaw Medical University, Marcinkowski Street 2-6, 50-368 Wroclaw, Poland; 3 Department of Angiology, Hypertension and Diabetology, Wroclaw Medical University, Borowska Street 213, 50-556 Wroclaw, Poland

**Keywords:** metabolic syndrome, DASH, diet, diet quality, visceral obesity

## Abstract

The current study was designed to examine the relationship between diet quality, as measured by the Dietary Approaches to Stop Hypertension (DASH) score, and the prevalence of metabolic syndrome (MetS) and its components. The study was cross-sectional in design and enrolled 535 people, including 215 with MetS and 320 without MetS. Using a validated food frequency questionnaire, the DASH diet quality score was counted. The mean age of the MetS group and control subjects was 58.48 ± 14.65 and 58.33 ± 9.26 years, respectively. The study showed that the MetS group had a significantly lower mean total DASH score than the control group (23.13 ± 5.44 vs. 24.62 ± 5.07, *p* = 0.0023). In addition, it was found that an increase in the total DASH score was associated with a lower risk of MetS (odds ratio [OR] = 0.95, 95% confidence interval [CI] = 0.91–0.99, *p* = 0.009). In the correlation analysis of the total population, a better-quality diet (higher DASH score) was positively correlated with high-density lipoprotein cholesterol (HDL-c), and negatively correlated with triglyceride (TG) concentration and waist circumference. It was also found that an increase in the total DASH score was associated with a lower risk of abdominal obesity (OR = 0.93, 95% CI = 0.88–0.99, *p* = 0.017). The results from the analyzed data highlight the potential benefits of following a healthy diet such as DASH.

## 1. Introduction

There is no single established dietary pattern for patients with metabolic syndrome (MetS) due to the varied components of the MetS in individual patients. Studies have used various diets and nutritional practices for MetS diet therapy, including low- and very low-fat diets (<30% or <10% energy from dietary fat), and low- and very low-carbohydrate diets (26–45% or <26% energy from dietary carbohydrates), as well as the Mediterranean diet (MD), DASH diet (Dietary Approaches to Stop Hypertension), MIND diet (Mediterranean-DASH Intervention for Neurodegenerative Delay), portfolio diet, vegan diet, vegetarian diet, Zone diet and Nordic diet [1,2,3]. Several dietary patterns may be helpful in the partial or complete reversal of metabolic disorders, particularly the Mediterranean, DASH, vegetarian, low-carbohydrate, and low-fat diets [4,5]. All of the proposed dietary patterns have a different effect on each of the MetS components and risk factors, including diabetes, hypertension, and cardiovascular disease (CVD) [1].

The benefits of a given dietary pattern must be combined with calorie restriction in the diet [1], as excessive body weight is widespread in people with MetS [6]. Therefore, many interventional studies have focused on reducing body weight, which have led to improvements in all MetS components [1]. In addition to environmental factors such as proper diet and weight control, physical activity is also important in the prevention and treatment of MetS [4,7,8].

The DASH diet is considered one of the healthiest diets in the world and was initially only used in patients with arterial hypertension and for preventing the development of arterial hypertension [9,10]. The main principles of the DASH diet plan are based on choosing foods that are low in saturated fatty acids and trans fats, high in potassium, magnesium, calcium, fiber, and protein, and low in sodium [11,12]. The DASH diet recommends eating vegetables, fruits, and whole-grain products. In addition, it is recommended to eat low-fat or fat-free dairy products, as well as fish, poultry, beans, nuts, and vegetable oils. In the DASH diet, products that are a source of saturated fatty acids, such as high fat and highly processed meat, full-fat dairy products, and oils such as coconut and palm, should be limited. Also, the consumption of sweetened beverages and sweets should be limited, as should sodium intake [11,12,13,14]. To meet the DASH diet plan goal of a 2000-calorie-a-day diet, daily food consumption should include 6–8 servings of grains and grain products, ≤2 servings of 3 ounces of meat, poultry and fish, 4–5 servings of vegetables, 4–5 servings of fruits, 2–3 servings of low-fat or fat-free dairy products, 2–3 servings of fats and oils, and ≤2300 mg of sodium (although ≤ 1500 mg of sodium lowers blood pressure (BP) even further than 2300 mg of sodium). Meanwhile, weekly food consumption should include 4–5 servings of nuts, seeds, dry beans, and peas and should be limited to ≤5 servings of sweets [11].

Many scientific studies have found following the DASH diet to be associated with a lower risk of colorectal cancer [15], heart disease [16], strokes [17], diabetes [18], and obesity [19]. In a systematic review and meta-analysis of the prospective studies, Soltani et al. [20] found that even modest adherence to the DASH diet was associated with a lower risk of all-cause and cause-specific mortality, including CVD, cancer, and stroke. The health benefits associated with the DASH diet, or a similar diet, may be related to the inclusion of a large number of antioxidants in the diet [9]. The DASH diet is currently used in the treatment of overweight and obese people, as well as individuals with MetS [21,22]. Indeed, the DASH diet is universally used for those with MetS, as demonstrated by numerous scientific studies [21,23]. Some researchers found that a greater adherence to the DASH diet in MetS patients was associated with a smaller waist circumference (WC) [19] and was negatively associated with elevated BP, high triglycerides (TGs), and low concentrations of high-density lipoprotein cholesterol (HDL-c) [24].

Meanwhile, many studies have found an inverse relationship between adherence to the DASH diet and the prevalence and progression of MetS [19,24,25], though not all studies have confirmed this relationship [26]. Further interventional studies are required to elucidate the exact effect of the DASH diet on MetS [27].

This study aimed to assess the cross-sectional association between adherence to the DASH diet and the prevalence of MetS and its individual components. The study investigated whether higher diet quality, as measured by the DASH score, was associated with a lower risk of MetS and its components. In addition, the study evaluated the correlations between the total DASH score, its components, and MetS components. Specifically, the research questioned if diet quality differed in patients with MetS compared to those in the control group. In addition, whether the content of individual components of the DASH score significantly differed in patients with MetS compared to those in the control group was assessed.

## 2. Materials and Methods

### 2.1. Study Design and Study Participants 

This study was a cross-sectional study in design, and the study group consisted of patients with MetS (the MetS group). They were recruited from the Clinical Department of Internal Diseases and the Clinical Department of Endocrinology in the 4th Military Hospital of Wroclaw. Nutrition interviews were collected from the MetS group between 2013 to 2017. The initial MetS group consisted of 222 patients, but 7 patients were excluded from the study (missing data and daily rations < 700 or >5000 kcal per day). This left a final MetS group that included 215 patients, of whom 127 were women and 88 were men. The MetS group had three or more components of MetS, according to the criteria defined by the American Heart Association (AHA), the National Heart, Lung, and Blood Institute (NHLBI), and the International Diabetes Federation (IDF) [28,29]. The control group data were obtained from the 6-year follow-up of the Prospective Urban and Rural Epidemiology (PURE) Poland study. Nutritional interviews and other data were collected for the control group from 2013–2016. The design, justification, and methodology of the PURE study were described by Teo et al. [30]. The control group consisted of people with less than three components of MetS, based on the same criteria used for the MetS group. It was noted that 746 subjects were not diagnosed with MetS. However, 422 people were excluded from the study due to incomplete data (lack of nutritional questionnaire, anthropometric measurements, and weight measurement). Those who provided unreliable information on daily food rations (<700 kcal or >5000 kcal per day) were also excluded from the study. The final control group consisted of 320 healthy people and was composed of 210 women and 110 men. The mean ages of the MetS and control groups were 58.48 ± 14.65 and 58.33 ± 9.26 years, respectively [31]. All participants provided written informed consent before participating in this study. 

The study was approved by the Bioethics Committee of Wroclaw Medical University (no. KB-306/2018).

### 2.2. Dietary Assessment

The eating habits of both groups were assessed using a 154-item semi-quantitative food frequency questionnaire (FFQ) that had been validated for the Polish population [32]. The validity and reliability of the FFQ were previously reported [32]. The questionnaire made it possible to collect the mean frequencies (daily, weekly, monthly) of food consumption in the MetS and control groups. For each food product in the FFQ, a serving size expressed in household measures was specified. The obtained data were transformed to estimate the mean daily intake. On this basis, the nutritional value of the daily food rations of the test and control groups was calculated using Food Composition Tables [33].

### 2.3. DASH Score

The DASH score was calculated for each person in the study based on the information collected through the FFQ. To neutralize the effect of energy intake on the eight DASH score components, each food group and food ingredient was calculated per 1000 kcal [34]. The DASH score was then computed according to the method of Fung et al. [17] which is based on foods and nutrients that are essential in the DASH diet and those that should be limited in the diet. The eight components of the DASH score encompass seven groups of food products and one nutrient, including a high intake of fruits, vegetables, nuts and legumes, whole-grain products, and low-fat dairy products, as well as a low intake of red and processed meat, sodium, and sweetened drinks. The respondents received a score of 5 when the consumption of fruits, vegetables, nuts and legumes, low-fat dairy products, and whole-grain cereals were in the highest quintile in the study population. In contrast, respondents received 1 point when the consumption of red and processed meat, sodium and sweetened beverages were in the largest quintile in the study population. As the population of the current study included both genders, intake rankings were assigned for each gender separately. The total number of points obtained for this indicator ranged from 8 to 40. Lower values of the total score indicated a poor-quality diet, while higher values indicated a better-quality diet [17].

### 2.4. Biochemical and Anthropometric Assessment

Information on TGs, HDL-C, and fasting glucose (FG) was collected from the results of the biochemical test, and information on medications taken was derived from the medical history of patients with MetS during hospitalization. For the control group, blood was drawn for biochemical tests, and BP and anthropometric measurements were taken during the PURE study. FG, TGs and HDL-c concentrations were measured in venous blood samples. TGs and HDL-c concentrations were measured using the SPRINREACT enzymatic assay (Sant Esteve De Bas, Girona, Spain), while FG levels were tested using the Ascensia Entrust Glucometer (Bayer AG, Leverkusen, Germany).

In both groups, BP was measured in a sitting and relaxed position after a 5-minute rest, using a certified digital sphygmomanometer (Omron HEM-711 IntelliSense, Tokyo, Japan). The mean BP values included in the analyses were derived from two measurements from each participant.

WC was measured at the midpoint between the lower marginal edge and the iliac crest, and measurements are expressed in centimeters with an accuracy of 0.5 cm. Body weight was measured with an accuracy of 0.1 kg and body height was measured with an accuracy of 0.5 cm. The body mass index (BMI) was calculated for both groups using the weight and height measurements (BMI = weight (kg)/height (m)^2^). The study and data collection details had been published previously [31].

### 2.5. Definition of Terms

The criteria for the clinical diagnosis of MetS used in the study were standardized in 2009 by representatives of the IDF, NHLBI, AHA, WHF (World Heart Federation), IAS (International Atherosclerosis Society), and IASO (International Association for the Study of Obesity) [28]. The WC criteria defining abdominal obesity were adopted for the European population recommended by the IDF [29]. The presence of three out of five components qualified people for a diagnosis of MetS, and included (1) abdominal obesity (WC ≥ 80 cm for women and ≥94 cm for men); (2) elevated TGs ≥ 150 mg/dL (or drug treatment for elevated TGs); (3) lowered HDL-c of <50 mg/dL in women and <40 mg/dL in men (or pharmacological treatment of lowered HDL-c); (4) elevated BP, with systolic blood pressure (SBP) ≥ 130 and/or diastolic blood pressure (DBP) ≥ 85 mm Hg (or pharmacological antihypertensive treatment); (5) elevated FG ≥ 100 mg/dL (or pharmacological treatment of elevated blood glucose) [28].

### 2.6. Statistical Analysis

The study assessed descriptive features depending on gender and the presence of MetS. The descriptive data are presented as mean and standard deviation for numeric variables. The normality of the distribution of continuous variables was assessed using graphical (a histogram) and analytical methods (the Shapiro-Wilk test). The Mann–Whitney U test was used to analyze subgroups of non-normally distributed variables, and Student’s *t*-test was used to compare means for normally distributed data.

Multivariate logistic regression analysis was performed to find significant determinants of the prevalence of Mets and its components, and included total DASH score, age, gender, BMI, the daily energy intake, and the taking of drugs to lower TGs, as independent variables (model I-VI). In the model in which the relationship of certain independent variables with low HDL-c and high TGs was examined, the use of drugs to lower TGs was not taken into account. Before building the logistic regression models, it was determined if the response variable was binary, whether there was a lack of collinearity among the explanatory variables using a correlation matrix, and whether there was a linear relationship between the continuous explanatory variables and the logit of the response variable. Only independent variables that met these assumptions were selected for the multivariate logistic regression model.

The relationships between the components of the DASH score expressed in grams or mg per 1000 kcal and the MetS components in the total population and within each gender group were analyzed using Kendall’s tau correlation.

The level of statistical significance was set at *p* < 0.05. The analyses were performed using Statistica v.13.3. (TIBCO Software Inc., Palo Alto, CA, USA).

## 3. Results

### 3.1. Descriptive Characteristics

Table 1 presents data on the characteristics and MetS components of all study participants based on gender and MetS status. The mean ages of the women and men in the Mets group were 58.57 ± 15.93 and 58.34 ± 12.66 years, respectively. In the control group, the mean age of the women was 59.04 ± 9.11 and the mean age of the men was 56.98 ± 9.44 years. Both women and men with MetS had significantly higher body weight than women and men from the control group. Also, the average BMI value in the women and men in the MetS group was significantly higher than in the women and men in the control group. Comparing the values of MetS components, the mean HDL-c concentrations were lower in the women and men from the MetS group. In comparison, the mean concentrations of TGs, FG, and WC were higher than in the women and men without MetS. However, there were no differences in mean SBP or DBP between the MetS and control groups for both sexes.

Table 2 presents the mean values of DASH score components for the MetS group and the control group, as well as for the women and men with and without MetS. The study showed that the MetS group had a significantly lower total DASH score than the control group. Women with MetS also had a significantly lower mean total DASH score than women without MetS, though there was no difference in the total DASH score in men. A significantly lower mean value for the total fruit intake was observed among the DASH score component in the MetS group compared to the control group. Women with MetS also had a lower mean total fruits intake than women without MetS, though this relationship was not found in men. However, a significantly lower intake of nuts and legumes was observed in the MetS group than in the control group and in the group of women and men with MetS, compared to women and men without MetS. On the other hand, higher sodium content in the diet was observed in the MetS group compared to the control group and in women and men with MetS compared to women and men without MetS. For red and processed meat, significantly higher intake was also found in the MetS group than in the control group. A similar relationship to this component of the DASH score was shown in the group of women with MetS compared to women without MetS. However, there was no significant statistical difference in red and processed meat between men from the MetS group and men from the control group. DASH score components for which no significant differences between groups were found included vegetables, whole grains, low-fat dairy products and sweetened beverages.

### 3.2. Multivariate Logistic Regression Analysis

Table 3 presents the results of the multivariate logistic regression model used to assess whether individual independent variables were associated with the prevalence of MetS. The model showed that taking drugs to lower the concentration of TGs and increasing BMI significantly increased the risk of MetS prevalence (TGs – odds ratio [OR] = 2.24, 95% confidence interval [CI] = 1.43–3.51, *p* < 0.0001; BMI – OR = 1.27, 95% CI = 1.21–1.33, *p* < 0.0001). It was also found that an increase in the total DASH score was significantly associated with a lower risk of MetS prevalence (OR = 0.95, 95% CI = 0.91–0.99, *p* = 0.009).

Table 4 presents the results of the multivariate logistic regression model used to assess the association of independent variables with the prevalence of MetS components. It was found that BMI was significantly associated with a risk of low HDL-c concentration (OR = 1.18, 95% CI = 1.14–1.23, *p* < 0.0001). However, an increase in the total DASH score was shown to reduce the risk of this MetS component, but the result was not statistically significant. It was also found that the risk of abnormally elevated TG values was significantly higher in men than in women (OR = 1.6, 95% CI = 0.93–0.99, *p* = 0.044). On the other hand, an increase in BMI was significantly associated with a higher risk of abnormal FG levels (OR = 1.15, 95% CI = 1.11–1.19, *p* < 0.001). Meanwhile, the risk of abdominal obesity substantially increased with an increase in BMI (OR = 2.38, 95% CI = 2.03–2.79, *p* < 0.0001) and significantly reduced with an increase in total DASH score (OR = 0.93, 95% CI = 0.88–0.99, *p* = 0.017). An increased risk of high BP was strongly associated with taking drugs to lower TGs (OR = 4.50, 95% CI = 2.30–8.82, *p* < 0.0001), the male sex (OR = 1.81, 95% CI = 1.12–2.92, *p* = 0.015), and an increase in BMI (OR = 1.18, 95% CI = 1.11–1.24, *p* < 0.0001).

### 3.3. Correlation Analysis

Table 5 presents the correlations between individual DASH score components, total DASH score, and individual MetS components for the total population. A better-quality diet (higher DASH score) was positively correlated with HDL-c and negatively correlated with TGs and WC. Meanwhile, a higher consumption of total fruits, nuts and legumes, and whole grains was positively correlated with HDL-c. However, a higher consumption of sodium and red and processed meat was negatively correlated with HDL-c. A higher total fruit intake was inversely associated with TGs, FG, WC, and DBP. A higher consumption of nuts and legumes was negatively correlated with TGs and WC, while a higher sodium intake correlated positively with TGs and WC. It was also shown that a higher consumption of red and processed meat was positively correlated with TGs, FG, and WC.

A better diet quality (a higher DASH score) was positively correlated with HDL-c and negatively correlated with TGs and WC in women. However, no correlation was found between the total DASH score and the individual MetS components in men. Among the DASH score components, a higher consumption of total fruits, nuts and legumes, and whole grains, was positively correlated with HDL-c in women. In contrast, a higher intake of sodium and red and processed meat was negatively correlated with HDL-c in women. Higher consumption of nuts and legumes was correlated positively with HDL-c and negatively with WC in men, while it was negatively correlated with TGs and WC in women. A higher consumption of total fruits was also negatively correlated with TGs and WC in women, although a higher consumption of vegetables correlated positively with WC. A higher consumption of whole grains was correlated negatively with WC and DBP in men, while a higher consumption of low-fat dairy products correlated positively with TGs. It was found in women that a higher sodium intake was correlated positively with TGs and WC. On the other hand, a higher consumption of red and processed meat was positively correlated with WC in women and with FG and WC in men. All significant correlations were weak and the highest r value was 0.168.

## 4. Discussion

This study assessed the relationship between diet quality measured using the DASH score and the prevalence of MetS and its components in 535 participants (215 patients with MetS and 320 without MetS) to determine if a diet similar to DASH was associated with a lower risk of MetS and its components.

The study found no significant difference in the energy value of daily food rations in the group of women with MetS compared to women without MetS. Similar statistically insignificant results were obtained in the group of men. The most effective intervention in people with MetS is limiting the energy intake of their diet. Indeed, changing nutrition to achieve a 7–10% weight loss is appropriate for people with prediabetes unless additional weight loss is desired for other purposes [1]. However, there is no universal dietary pattern that is recommended for MetS patients, meaning that their dietary recommendations may be based on their metabolic goals, socioeconomic factors, food availability, and personal and cultural preferences [1].

One of the proposed nutritional interventions for MetS is the DASH diet. The DASH diet emphasizes greater consumption of fruits, vegetables, nuts, legumes, whole-grain cereals, and reduced-fat dairy products. Furthermore, it limits the consumption of red meat, sweetened beverages, sugar, total fats, and saturated fatty acids [17]. This study showed a lower mean total DASH score for the MetS group compared to the control group, as well as for women with MetS compared to women without MetS.

Diet can contribute to reducing the risk of prevalence and the deterioration of MetS and its components [1,35]. Diets high in monounsaturated fatty acids (MUFAs) are recommended for MetS patients because they improve the lipid panel and increase insulin sensitivity compared to saturated fatty acids (SFAs) [1]. Low-glycemic index (low-GI) diets are also recommended for this group of patients. Diets with a low-GI, which are a source of fiber, increase the feeling of satiety, reduce insulin resistance, and decrease the risk of developing diabetes [1]. Among dietary factors, a high consumption of fruits, vegetables, legumes, and nuts, and a low consumption of high-fat dairy products, red and processed meat, and sodium, reduces the risk of MetS and its components [36,37]. In the herein study, the entire MetS group and women with MetS had a lower mean intake of total fruits and a higher mean content of red and processed meat in their diet compared to the mean content of the DASH score components in the control group and women without MetS, respectively. In addition, the MetS group and the women and men with MetS had a significantly lower mean intake of nuts and legumes and a higher mean content of sodium in their diets compared to the control group and women and men without MetS. To improve diet quality and increase adherence to the DASH diet in patients with MetS, it is worth increasing the supply of fruits, nuts, and legumes in their daily rations. One serving of fruit is equivalent to 1 medium fruit (such as apples, pears, and bananas), a ¼ cup of dried fruit (raisins for example), or ½ cup of fresh or frozen fruit (grapes, strawberries, and raspberries). One serving of nuts and legumes is equivalent to 1/3 cup of nuts (almonds, hazelnuts, mixed nuts, peanuts, and walnuts), 2 tablespoons (Tbsps) of peanut butter, 2 Tbsps of seeds (sunflower seeds for example), or a ½ cup of cooked legumes (kidney beans, split peas). On the other hand, food products that are a source of sodium; red and processed meat such as sausages, pork belly, head cheese; and luncheon meat should be kept to a minimum.

This study found that an increase in the total DASH score significantly reduced the risk of MetS and was associated with a lower risk of abdominal obesity. In a study by Ghorabi et al. [24], an inverse relationship was found between adherence to the DASH diet and the likelihood of MetS (OR = 0.25, 95% CI = 0.15–0.42, *p* < 0.001). Also, after adjusting the model for energy intake, socioeconomic status, and BMI, participants in the highest tertile of DASH scores were 72% less likely to have MetS compared to the lowest tertile of DASH scores (OR = 0.28, 95% CI = 0.14–0.54, *p* < 0.001). In addition, Ghorabi et al. [24] found that adherence to the DASH diet was inversely related to the likelihood of elevated BP (OR = 0.09, 95% CI = 0.04–0.17, *p* < 0.001), elevated serum TGs (OR = 0.41, 95%CI = 0.24–0.70, *p* = 0.001) and reduced HDL-c (OR = 0.32, 95% CI = 0.18–0.57, *p* < 0.001). In their adjusted model, adherence to the DASH diet was inversely related to elevated BP (OR = 0.12, 95% CI = 0.05–0.29, *p* < 0.001), while the results for high TGs (OR = 0.53, 95% CI = 0.28–1.00, =0.049) and low serum HDL-C (OR = 0.51, 95% CI = 0.25–1.01, *p* = 0.053) were at the limit of statistical significance. However, they did not find a relationship between adherence to the DASH diet and abdominal obesity. Furthermore, in their fully adjusted model, there was no significant association between adherence to the DASH diet and elevated FG. Piment et al. [38] also assessed the relationship between adherence to the DASH diet and the risk of MetS. In their model, after adjusting for age, gender, smoking, alcohol consumption, physical activity, and BMI, higher dietary adherence to the DASH diet was not associated with the risk of MetS (risk ratio [RR] = 0.82; 95% CI = 0.64–1.03; *p* = 0.083). However, analyses by tertile of alcohol consumption showed that greater adherence to the DASH diet was inversely related to the risk of MetS in people with low alcohol consumption (RR = 0.41; 95% CI = 0.20–0.85; *p* = 0.023).

In the current study, a higher DASH score was positively correlated with HDL-c concentration, while it was negatively correlated with TG concentration. WC. Millar et al. [39] assessed the relationship between total DASH score, Healthy Eating Index-2015 (HEI-2015), MD, and Energy-adjusted Dietary Inflammatory Index™ (E-DII™), and plasma lipid, and lipoprotein panels, to test the hypothesis that a healthier diet (its better quality and more anti-inflammatory effects) would be associated with a more favorable lipoprotein profile. The study involved 1862 people aged 46–73. The study found that a higher total DASH score (better diet quality) was positively correlated with HDL-c (r = 0.175, *p* < 0.001), large LDL (r = 0.150, *p* < 0.001), total HDL-c (r = 0.052, *p* = 0.012), large HDL-c (r = 0.185, *p* < 0.001) and medium HDL-c (r = 0.065, *p* = 0.006). On the other hand, it was negatively correlated with TGs (r = −0.124, *p* < 0.001), total triglyceride-rich lipoprotein (TRL) (r = −0.099, *p* < 0.001), large very low-density lipoprotein (VLDL) (r = −0.150, *p* < 0.001), medium VLDL (r = −0.135, *p* < 0.001), total low-density lipoprotein (LDL) (r = −0.083, *p* < 0.001), intermediate-density lipoprotein (IDL) (r = −0.083, *p* <0.001), small LDL (r = −0.171, *p* <0.001) and small HDL (r = −0.143, *p* < 0.001). The study also found that lipoprotein concentrations were more strongly correlated with the DASH diet than the HEI-2015, MD, or E-DII™ scores.

In the study by Millar et al. [39], linear regression analysis was used to describe the relationships between the values of individual indices evaluating the quality of the diet and plasma lipids as well as the concentrations and sizes of lipoprotein particles. The researchers ran two models, the first of which was adjusted for sex and age, and the second model was adjusted for sex, age, education, cholesterol-lowering medication use, type 2 diabetes, smoking, physical activity, BMI, and total energy intake. In the fully adjusted model (model 2), the authors showed that a higher diet quality or a more anti-inflammatory diet was associated with lower total cholesterol (DASH − β = −0.072, *p* = 0.006; HEI-2015 − β = −0.052, *p* = 0.037), TGs (DASH − β = −0.025, *p* = 0.036) and LDL-c (DASH − β = −0.052, *p* = 0.022; E-DII − β = 0.046, *p* = 0.033) [39]. However, no significant relationship was observed for HDL-c. They also demonstrated that the DASH diet indicator may be a better marker of improved cardiometabolic health (characterized by a less proatherogenic cardiometabolic profile) than the other indicators of diet quality they analyzed [39]. Furthermore, in the Nurses’ Health Study, which involved 775 healthy women, it was found that people with better diet quality, measured by DASH or MD indices, had lower concentrations of TGs compared to people with lower diet quality [40].

In a study by Philips et al. [41], the relationship between diet quality, measured by the DASH score, and biomarkers of cardiometabolic health was examined. In their study, higher diet quality was associated with a lower BMI (*p* < 0.05), and WC (*p* < 0.001), as well as lower levels of tumor necrosis factor-alpha (TNF-alpha), interleukin 6 (IL-6), and plasminogen activator inhibitor-1 (PAI-1) (*p* < 0.01), a lower white blood cell count (WBC) (*p* < 0.01) and reduced insulin resistance (*p* < 0.05). In addition, individuals in the highest DASH quartile were 54% and 48% less likely to develop central obesity and MetS, respectively, than those in the lowest quartile (*p* < 0.05).

Studies have proposed the mechanisms behind the beneficial effects of a higher adherence to the DASH diet on MetS risk, although they are not yet clearly understood [27]. Higher intakes of fiber, folic acid, potassium, magnesium, calcium, vitamin C, and phytochemicals such as phytosterols, carotenoids, and flavonoids from the DASH diet may have beneficial effects on BP, lipid profile, insulin sensitivity, and total antioxidant capacity, all of which reduce the risk of MetS [27].

Although the DASH diet was first introduced more than 20 years ago, it is still the subject of numerous scientific studies, and the use of the DASH diet is still relevant and reasonable [14,42]. In addition, the DASH diet is considered an important advancement in nutritional science [42]. Due to the health benefits of the DASH diet, it is an integral part of national BP and dietary guidelines [42]. However, in the US, where the DASH diet was initiated, adherence to the DASH diet is still poor [43,44]. Steinberg et al. [42] concluded that poor adherence to the DASH diet is related to the high price of fruits and vegetables in the US and cheap and readily available highly processed foods. Nonetheless, a study by Young et al. [45] showed that the DASH diet can also be followed by those on a low-income and claimed that dried beans and frozen vegetables are inexpensive and are in accordance with the DASH diet. According to Steinberg et al. [42], the main public health challenge is the continued dissemination and implementation of the DASH diet in practice. In this regard, it is hoped that this cross-sectional study will spur the creation of a digital health tool for Polish patients with MetS and that uptake of the DASH diet will be increased through its dissemination.

This study found that higher diet quality was associated with a lower risk of MetS and visceral obesity, and was positively correlated with HDL-c and inversely correlated with TGs and WC. This cross-sectional study may provide a basis for designing longitudinal studies to examine these associations in the Polish population. In clinical practice, the DASH diet can be proposed as one of the most important components of lifestyle change for the prevention of MetS in at-risk individuals as well as patients with MetS. The need for the study stemmed from the fact that there are only a few scientific studies on the diet quality of patients with MetS in Poland. Patients with MetS are at risk of CVD and CVD-related death. Therefore, increasing the awareness of clinicians in Poland about a holistic approach to diet and the implementation of dietary changes for patients with MetS, as well as monitoring the quality of their diet, could significantly improve the quality of life of these patients.

This study had several strengths. As far as we know, this is the first study to compare the DASH score with the prevalence of MetS and its components in the Polish population. Research based on a specific indicator of dietary quality is important from a public health perspective as it provides greater insight into the causes of chronic diseases and MetS.

The study also had several limitations similar to those described in our previous study [31]. The study population was recruited from the Lower Silesian Voivodeship in Poland. Therefore, the translation of the results to the entire adult population in Poland should be conducted with caution. In addition, the multivariate logistic regressions and their resultant ORs were not adjusted for confounders such as socioeconomic status, physical activity, sedentary behavior, smoking, alcohol consumption, comorbidities, or family history.

## 5. Conclusions

Assessment of the whole diet takes into account the interactions of nutrients and their synergistic effects on the risk of chronic diseases and MetS. The results of this study suggest that higher diet quality, measured by the DASH score, is associated with a lower risk of MetS and one of its components, visceral obesity. The results from the analyzed data highlight the potential benefits of following a healthy diet such as DASH.

## Figures and Tables

**Table 1:** Characteristics of the study and control groups, stratified by gender and diagnosis of the metabolic syndrome.

	Women with MetS (*n* = 127)	Women without MetS (*n* = 210)	*p*-Value	Men with MetS (*n* = 88)	Men without MetS (*n* = 110)	*p*-Value
Characteristic	Mean score ± SD			Mean score ± SD		
Age [years]	58.57 ± 15.93	59.04 ± 9.11	0.900	58.34 ± 12.66	56.98 ± 9.44	0.164
Body weight [kg]	82.50 ± 20.19	66.44 ± 11.85	<0.0001	97.68 ± 19.36	78.81 ± 10.20	<0.0001
BMI [kg/m^2^]	32.02 ± 6.80	25.95 ± 4.60	<0.0001	32.08 ± 5.40	26.21 ± 3.01	<0.0001
Energy value of a daily food ration [kcal]	1879.72 ± 694.24	1908.87 ± 709.97	0.623	2140.86 ± 671.94	2018.13 ± 677.06	0.163
Metabolic syndrome components	Mean score ± SD		*p*-value	Mean score ± SD		*p*-value
HDL-c (mg/dL)	46.53 ± 15.89	71.24 ± 18.64	<0.0001	36.32 ± 11.93	58.76 ± 14.43	<0.0001
TGs (mg/dL)	153.17 ± 74.20	94.32 ± 35.42	<0.0001	178.30 ± 107.54	94.85 ± 32.56	<0.0001
Glucose (mg/dL)	109.25 ± 43.40	89.90 ± 8.31	0.0034	128.07 ± 48.72	91.81 ± 8.22	<0.0001
Waist circumference (cm)	100.71 ± 14.50	80.82 ± 11.15	<0.0001	111.06 ± 13.89	90.79 ± 8.84	<0.0001
Systolic blood pressure (mm Hg)	130.46 ± 17.88	131.76 ± 16.59	0.442	138.32 ± 17.91	137.33 ± 15.04	0.762
Diastolic blood pressure (mm Hg)	82.22 ± 11.22	81.48 ± 10.05	0.597	86.25 ± 10.78	85.70 ± 8.16	0.898

MetS—metabolic syndrome; SD—standard deviation; BMI—body mass index; HDL-c—high-density lipoprotein cholesterol; TGs—triglycerides.

**Table 2:** DASH component scores for MetS group and control group and women and men with and without MetS.

	MetS Group (*n* = 215)	Control Group (*n* = 320)	*p* Value	Women with MetS (*n* = 127)	Women without MetS (*n* = 210)	*p* Value	Men with MetS (*n* = 88)	Men without MetS (*n* = 110)	*p* Value
DASH components	Mean score ± SD			Mean score ± SD			Mean score ± SD		
Total fruits (g/1000 kcal)	156.68 ± 92.79	181.36 ± 108.81	0.012	162.96 ± 89.82	206.25 ± 115.30	0.001	147.62 ± 96.72	133.85 ± 75.42	0.606
Vegetables (except potatoes and legumes) (g/1000 kcal)	203.58 ± 84.68	196.42 ± 98.30	0.124	218.61 ± 93.33	206.07 ± 98.95	0.106	181.90 ± 64.93	177.99 ± 94.77	0.219
Nuts and legumes (g/1000kcal)	12.53 ± 14.55	16.89 ± 13.93	<0.0001	12.11 ± 13.12	17.50 ± 15.21	<0.0001	13.15 ± 16.46	15.72 ± 11.05	0.007
Whole grains (g/1000 kcal)	29.54 ± 31.30	29.69 ± 26.52	0.138	30.79 ± 30.33	30.21 ± 24.27	0.278	27.74 ± 32.73	28.69 ± 30.45	0.484
Low-fat dairy (g/1000 kcal)	92.06 ± 93.46	83.40 ± 90.72	0.225	100.40 ± 104.82	92.16 ± 102.78	0.340	80.04 ± 72.99	66.68 ± 58.32	0.359
Sodium (mg/1000 kcal)	1124.58 ± 306.09	1027.98 ± 264.65	0.001	1128.55 ± 306.56	1035.58 ± 268.69	0.005	1118.86 ± 307.07	1013.47 ± 257.33	0.049
Red and processed meat (g/1000 kcal)	36.76 ± 26.05	28.92 ± 20.63	0.0004	34.06 ± 23.58	26.78 ± 20.81	0.004	40.67 ± 28.95	33.02 ± 19.73	0.083
Sweetened beverages (g/1000 kcal)	72.17 ± 93.88	57.08 ± 67.86	0.581	72.61 ± 95.08	56.37 ± 61.47	0.626	71.53 ± 92.66	58.44 ± 78.92	0.767
Total DASH score	23.13 ± 5.44	24.62 ± 5.07	0.002	22.94 ± 5.45	24.69 ± 5.04	0.005	23.42 ± 5.45	24.49 ± 5.13	0.177

**Table 3:** Multivariate logistic regression analysis, odds ratios and 95% confidence intervals for the prevalence of MetS.

	Variables	Coefficient	SE	*p*-Value	OR	95% CI
Model I	TGs medication	0.807	0.228	<0.0001	2.24	1.43–3.51
	BMI	0.238	0.025	<0.0001	1.27	1.21–1.33
	DASH score	−0.053	0.020	0.009	0.95	0.91–0.99

SE—standard error; OR—odds ratio; CI—confidence interval; TGs—triglycerides; BMI—body mass index.

**Table 4:** Multivariate logistic regression analysis, odds ratios, and 95% confidence intervals for the prevalence of individual components of MetS.

		Variables	Coefficient	SE	*p*-Value	OR	95% CI
Model II	Low HDL-c	Gender (Ref. Women)	0.299	0.204	0.142	1.35	0.90–2.01
		BMI	0.166	0.020	<0.0001	1.18	1.14–1.23
		DASH score	−0.034	0.019	0.073	0.97	0.93–1.00
Model III	High TGs	Gender (Ref. Women)	0.470	0.191	0.014	1.60	1.10–2.32
Model IV	High blood glucose	Gender (Ref. Women)	0.382	0.203	0.060	1.47	0.98–2.18
		TGs medication	0.327	0.217	0.132	1.39	0.91–2.13
		BMI	0.139	0.019	<0.001	1.15	1.11–1.19
Model V	Visceral obesity	TGs medication	0.528	0.349	0.131	1.70	0.86–3.36
		BMI	0.867	0.080	<0.0001	2.38	2.03–2.79
		DASH score	−0.071	0.030	0.017	0.93	0.88–0.99
Model VI	Hypertension	Gender (Ref. Women)	0.593	0.244	0.015	1.81	1.12–2.92
		TGs medication	1.504	0.343	<0.0001	4.50	2.30–8.82
		BMI	0.160	0.027	<0.0001	1.18	1.11–1.24

SE—standard error; OR—odds ratio; CI—confidence interval; HDL-c—high-density lipoprotein cholesterol; TGs—triglycerides; BMI—body mass index.

**Table 5:** Tau Kendall correlations between DASH components, total DASH score, and metabolic syndrome components.

		HDL-c (mg/dL)	TGs (mg/dL)	FG (mg/dL)	WC (cm)	SBP (mm Hg)	DBP (mm Hg)
Total (*n* = 535) **	Total fruits (g/1000 kcal)	0.117 *	−0.062 *	−0.079 *	−0.117 *	−0.025	−0.059 *
	Vegetables (except potatoes and legumes) (g/1000 kcal)	0.018	0.034	−0.017	0.036	0.002	−0.003
	Nuts and legumes (g/1000 kcal)	0.126 *	−0.117 *	−0.040	−0.110 *	0.004	−0.015
	Whole grains (g/1000 kcal)	0.063 *	−0.027	−0.007	−0.051	−0.012	−0.053
	Low-fat dairy (g/1000 kcal)	−0.031	0.045	0.028	0.034	0.003	−0.035
	Sodium (mg/1000 kcal)	−0.064 *	0.078 *	0.030	0.102 *	−0.040	−0.034
	Red and processed meat (g/1000 kcal)	−0.106 *	0.075 *	0.072 *	0.168 *	−0.018	0.027
	Sweetened beverages (g/1000 kcal)	−0.028	0.021	0.011	−0.002	−0.027	0.029
	DASH total score	0.088 *	−0.081 *	−0.028	−0.081 *	0.035	−0.017
Women (*n* = 337)	Total fruits (g/1000 kcal)	0.095 *	−0.087 *	−0.049	−0.083 *	0.004	−0.036
	Vegetables (except potatoes and legumes) (g/1000 kcal)	−0.022	0.034	−0.034	0.095 *	0.007	0.044
	Nuts and legumes (g/1000 kcal)	0.125 *	−0.134 *	−0.010	−0.126 *	−0.015	−0.020
	Whole grains (g/1000 kcal)	0.074 *	−0.034	−0.008	0.002	0.032	−0.011
	Low-fat dairy (g/1000 kcal)	−0.019	0.009	0.018	0.032	0.019	−0.030
	Sodium (mg/1000 kcal)	−0.090 *	0.104 *	0.013	0.143 *	−0.022	0.004
	Red and processed meat (g/1000 kcal)	−0.091 *	0.053	0.025	0.140 *	−0.083 *	0.006
	Sweetened beverages (g/1000 kcal)	−0.016	0.002	−0.010	−0.009	−0.027	0.034
	DASH total score	0.112 *	−0.111 *	−0.009	−0.093 *	0.035	−0.029
Men (*n* = 198)	Total fruits (g/1000 kcal)	0.017	−0.006	−0.041	−0.028	0.010	0.008
	Vegetables (except potatoes and legumes) (g/1000 kcal)	−0.011	0.061	0.072	0.053	0.052	−0.014
	Nuts and legumes (g/1000 kcal)	0.148 *	−0.094	−0.093	−0.120 *	0.040	0.000
	Whole grains (g/1000 kcal)	0.022	−0.012	0.004	−0.117 *	−0.062	−0.108 *
	Low-fat dairy (g/1000 kcal)	−0.074	0.121 *	0.076	0.090	−0.006	−0.024
	Sodium (mg/1000 kcal)	−0.045	0.040	0.053	0.040	−0.065	−0.093
	Red and processed meat (g/1000 kcal)	−0.046	0.093	0.099 *	0.125 *	0.042	−0.008
	Sweetened beverages (g/1000 kcal)	−0.060	0.055	0.055	0.010	−0.041	0.011
	DASH total score	0.080	−0.041	−0.063	−0.076	0.037	0.010

* Significant r values (*p* < 0.05); ** Total—both the MetS group and control group; HDL-c—high-density lipoprotein cholesterol; TGs—triglycerides; FG—fasting glucose; WC—waist circumference; SBP—systolic blood pressure; DBP—diastolic blood pressure. Values are presented as Kendall’s tau correlation coefficients between the DASH diet quality index score and its individual components, and the values or concentrations of individual MetS components among the total population and in women and men. For the DASH, lower scores represent poorer and higher scores represent better quality diet.

## Data Availability

Not applicable.
